# Evaluation of AI Tools Versus the PRISMA Method for Literature Search, Data Extraction, and Study Composition in Glaucoma Systematic Reviews: Content Analysis

**DOI:** 10.2196/68592

**Published:** 2025-09-05

**Authors:** Laura Antonia Meliante, Giulia Coco, Alessandro Rabiolo, Stefano De Cillà, Gianluca Manni

**Affiliations:** 1 Department of Clinical Sciences and Translational Medicine, University of Rome Tor Vergata, Facoltà di Medicina e Chirurgia East Tower—6th Floor Via Montpellier 1, Rome, 00133, Italy, 39 3484772028; 2 Department of Ophthalmology, University Hospital Maggiore della Carita', Novara, Italy; 3 IRCCS—Fondazione Bietti, Rome, Italy

**Keywords:** artificial intelligence, systematic literature review, AI-assisted academic writing, AI-assisted data analysis, AI in systematic reviews, Connected Papers, Elicit, JenniAI, ChatPDF, AI, SLR

## Abstract

**Background:**

Artificial intelligence (AI) is becoming increasingly popular in the scientific field, as it allows for the analysis of extensive datasets, summarizes results, and assists in writing academic papers.

**Objective:**

This study investigates the role of AI in the process of conducting a systematic literature review (SLR), focusing on its contributions and limitations at three key stages of its development, study selection, data extraction, and study composition, using glaucoma-related SLRs as case studies and Preferred Reporting Items for Systematic reviews and Meta-Analyses (PRISMA)-based SLRs as benchmarks.

**Methods:**

Four AI platforms were tested on their ability to reproduce four PRISMA-based, glaucoma-related SLRs. We used Connected Papers and Elicit to perform research of relevant records; then we assessed Elicit and ChatPDF’s ability to extract and organize information contained in the retrieved records. Finally, we tested Jenni AI’s capacity to compose an SLR.

**Results:**

Neither Connected Papers nor Elicit provided the totality of the results found using the PRISMA method. On average, data extracted from Elicit were accurate in 51.40% (SD 31.45%) of cases and imprecise in 13.69% (SD 17.98%); 22.37% (SD 27.54%) of responses were missing, while 12.51% (SD 14.70%) were incorrect. Data extracted from ChatPDF were accurate in 60.33% (SD 30.72%) of cases and imprecise in 7.41% (SD 13.88%); 17.56% (SD 20.02%) of responses were missing, and 14.70% (SD 17.72%) were incorrect. Jenni AI’s generated content exhibited satisfactory language fluency and technical proficiency but was insufficient in defining methods, elaborating results, and stating conclusions.

**Conclusions:**

The PRISMA method continues to exhibit clear superiority in terms of reproducibility and accuracy during the literature search, data extraction, and study composition phases of the SLR writing process. While AI can save time and assist with repetitive tasks, the active participation of the researcher throughout the entire process is still crucial to maintain control over the quality, accuracy, and objectivity of their work.

## Introduction

Systematic literature reviews (SLRs) represent the highest level of evidence in scientific literature; therefore, playing a vital role in the field of research and academia [1]. The main purpose of SLR is to analyze and synthesize all the existing evidence related to a specific research topic, by adopting a methodical and rigorous manner, in order to minimize subjective judgments and provide a systematic and transparent approach [1,2]. The Preferred Reporting Items for Systematic reviews and Meta-Analyses (PRISMA) framework is widely used for conducting and reporting systematic reviews and meta-analyses in the health care field. It provides guidelines to ensure transparent and complete reporting, thereby facilitating assessment of study quality [3]. The method consists of a 27-item checklist and a flow diagram; its key components include title or abstract, introduction, methods, results, discussion, conclusion, and funding disclosure, all aimed at emphasizing transparency, completeness, and reproducibility in reporting systematic reviews and meta-analyses [3]. Nevertheless, the process of writing an SLR is complex and presupposes mastery of a working methodology that may be elaborate and time-consuming.

Artificial intelligence (AI) is becoming increasingly popular in the scientific field, as it can provide a fast and easy way to analyze large volumes of data and review records, summarize results, and assist in writing academic papers.

Generative AI refers to a subset of AI technologies that can generate new content, such as text, images, music, speech, video, or code, based on learning to predict the next word or sequence of words given the preceding context [4]. Large language models (LLMs) are a subset of generative AI that specifically focus on text generation, using deep learning algorithms to process and analyze data for complex pattern recognition and prediction [4,5]. They are considered generative because they can construct full sentences, paragraphs, or documents that did not exist before, effectively “generating” new content based on their understanding of language patterns and structures learned during their training phase [5].

In the context of medicine, generative AI and LLMs can be used for a variety of purposes, including assisting in medical research by reviewing and summarizing large volumes of literature [4].

One of the key challenges for LLM and generative models is the ethical use of AI and the potential for biases in the underlying training data; incomplete or inaccurate datasets can lead to unreliable results [5]. This would cause the loss of the ultimate purpose of the SLR, which is to give a complete and objective overview of the literature. Therefore, it is crucial to acknowledge the downsides of relying completely on AI in the process of scientific writing and conveying information.

This study aims to investigate whether and how AI may support the writing of an SLR, by evaluating its potential advantages at three key stages: literature search, data organization and extraction, and drafting of the final study. Using glaucoma as a case study, a field in which the authors have domain expertise, we aimed to provide a guide on how some of the most popular AI software used in the scientific field operates. Our goal was to illustrate their possible contributions and current limitations in assisting with SRL composition.

## Methods

### AI Tools Overview

Four of the most popular AI platforms currently used in academic scientific research (Elicit [6], Connected Papers [7], ChatPDF [8], and Jenny AI [9]) were tested and compared in their ability to perform research on four already published, glaucoma-related SLRs.

Elicit [6] is an AI tool that provides the function of researching papers of interest by entering keywords in the research box. The platform provides a summary of the “top 8 papers” identified. The studies are presented in the form of a list, with a little summary shown alongside, which can be useful in the selection process. Records can either be selected or deleted from the list and be sorted by publication date, number of citations, and title.

Filters can be applied for refining the results, which are restricted to specific criteria like publication date and study design, encompassing randomized controlled trials, longitudinal studies, reviews, and meta-analyses.

Elicit offers the possibility to upload PDFs and organize their information according to criteria of interest (main findings, outcome measured, intervention effect, study design, study count, duration, length of follow-up, patients’ sex and age, etc); moreover, authors can customize columns according to their preferences and include instructions for data presentation and any additional necessary [6].

Connected Papers [7] is a tool able to provide a visual representation of relevant research papers based on specific keywords, which can be entered in the research box [7]. It uses Semantic Scholar as a database to identify papers of interest; results will appear in the form of a list. Connected Papers does not offer the option to apply filters for refining the results, such as publication date and study design. Once a record of choice has been selected within the list of results, Connected Papers creates a visual graph, based on studies’ similarities with regard to citations and references [7,10]. The generated graph places related papers close to each other, with the more recent ones shown in a darker tone and the most cited ones displayed in larger sizes. The results are also represented as a list.

ChatPDF [8] is an AI-powered tool that can analyze and extract relevant information from research papers in PDF format. Records can be uploaded and stored in the ChatPDF library, and folders can be created to organize them [8]. We created four folders, one for each SLR included in our investigation. Queries can be directed at individual records or entire folders. Our investigation found that posing questions to a specific paper yielded more accurate responses compared to asking questions to all papers within a folder, where answers were frequently incomplete or inaccurate. As a result, we chose to direct queries toward single papers individually.

Jenny AI [9] is a “writing assistant” designed to assist researchers in academic paper writing. The writing process is automated, using AI algorithms to generate content based on the input provided [9]. The “writing assistant” feature uses natural language processing algorithms to optimize the text and suggest improvements in word choice [9]. Given detailed instructions on what to write, Jenny is able to write entire paragraphs, such as introduction and conclusion; moreover, we can ask Jenny to write an opposing argument or write with more depth [9]. A paragraph can be selected, and we can edit it by asking to summarize it or make it longer, simplify what has been written, improve fluency, translate, or paraphrase in the linguistic register of our choice. Additionally, it enables the creation of content based on selected PDFs, facilitating the writing of tailored material [9].

In order to test the above-cited AI tools, we evaluated four already published, glaucoma-related SLRs. The initial analysis was focused on the review entitled “Glaucoma Pseudoexfoliation and Hearing Loss: A Systematic Literature Review” which we authored [11]. Subsequently, our research expanded to incorporate three additional SLRs. These were identified by conducting research in the journal “*Survey of Ophthalmology.*” We used “glaucoma” as a keyword filter and sorted results by publication date, starting from the most recent. We then selected the first three SLRs that the research produced. The first one focused on assessing the “Efficacy of Ahmed and Baerveldt glaucoma drainage device implantation in the pediatric population: A systematic review and meta-analysis” [12]. The second one analyzed “Standalone XEN45 Gel Stent implantation in the treatment of open-angle glaucoma: A systematic review and meta-analysis” [13], and the third one focused on “Nutritional supplementation in the treatment of glaucoma: A systematic review” [14]. The research was conducted on February 15, 2023.

### Literature Search

We first tested the ability of the AI software, Connected Papers [7] and Elicit [6], to identify studies of interest for our investigation. Using keywords specific to each systematic review of interest, searches on both platforms were conducted, mirroring the approach used in traditional databases like PubMed [15] or Embase [16]. When available, we used the exact search terms specified by the authors. The keywords applied were: “Glaucoma pseudoexfoliation and hearing loss” for the first SLR of interest [11]; “Efficacy of Ahmed and Baerveldt glaucoma drainage device in pediatric population” for the second SLR [12]; “Standalone XEN45 Gel Stent implantation in the treatment of open-angle glaucoma” for the third [13]; “Nutritional supplementation in the treatment of glaucoma” for the last evaluated SLR [14]. We compared the findings obtained from Elicit [6] and Connected Papers [7] with the studies included in each of the four SLRs under study, which were carried out using the PRISMA method. Our evaluation focused exclusively on the identification phase of the PRISMA [3] framework, assessing how well the AI tools retrieved relevant studies based on these keywords. Subsequent PRISMA [3] stages, such as records’ screening, eligibility, or inclusion, were not evaluated. The goal was to determine the effectiveness of Elicit [6] and Connected Papers [7] in replicating or streamlining the initial identification step typically performed in systematic reviews.

### Data Extraction

We explored the potential of Elicit and ChatPDF by testing their ability to extract and organize information contained in the records’ text, thus facilitating the understanding of the content of scientific studies. In order to obtain these results, all papers contained in each review were uploaded into two of the examined software tools, Elicit and ChatPDF, and asked them to create the same tables as the ones presented in the SLRs of our interest. Subsequently, the generated results were compared with the original tables presented in the SLRs, categorizing the obtained results as follows: “accurate” for responses that were both appropriate and precise, “imprecise” for responses that lacked critical details specified by the original authors, “missing” for instances where no response was provided, and “incorrect” when a response was factually inexact.

### AI-Assisted Writing

We tested Jenni AI’s ability to write an SLR by simulating various writing scenarios and assessing its efficacy. We gave Jenny specific instructions on writing an SLR with the purpose of reproducing the four reviews of interest. We asked Jenni to “write an SLR on glaucoma, pseudoexfoliation, and hearing loss,” for the first SLR of interest [11]; “write an SLR on the Efficacy of Ahmed and Baerveldt glaucoma drainage device in pediatric population,” for the second SLR [12]; “write an SLR on Standalone XEN45 Gel Stent implantation in the treatment of open-angle glaucoma” for the third [13]; “write an SLR on nutritional supplementation in the treatment of glaucoma” for the last analyzed SLR [14]. To ensure precise and relevant outputs, we used Jenni AI’s “Library” feature by uploading the records included in each of the SLRs of interest into the system. This enabled Jenni AI to tailor the generated content based on these specific studies and ensured that the citations provided were accurate and directly tied to the relevant studies. Two authors (LAM and GM) independently evaluated the generated content, focusing on language fluency, technical proficiency, suitability of the content, appropriateness and number of citations, defined methods (definition of research terms, inclusion and exclusion criteria, definition of data extraction, and risk of bias assessment), results (number of records identified, number of records excluded, description of each selected study, synthesis of results, statistical analysis, and risk of bias), discussion (interpretation of results), and conclusions. Each item was evaluated for the purpose of an SLR using a scale of 1=poor to 5=excellent. The two authors also measured the time used to write the paper using Jenni AI.

### Ethical Considerations

This study is based entirely on secondary analysis of publicly available scientific literature and publicly accessible AI-based tools; it did not involve human participants, identifiable personal data, or any form of medical or behavioral intervention. Therefore, ethical approval was not required.

## Results

### Literature Search

#### Connected Papers

The research of relevant records on the first SLR, “Glaucoma pseudoexfoliation and hearing loss” produced 22 results, which included 7 of 30 papers selected with the PRISMA method for our SLR, and the review of interest [11].

The second review, “Efficacy of Ahmed and Baerveldt glaucoma drainage device implantation in the pediatric population: A systematic review and meta-analysis,” included 32 studies. None of the original 32 records were identified by Connected Papers [12]. The third review, “Standalone XEN45 Gel Stent implantation in the treatment of open-angle glaucoma: A systematic review and meta-analysis,” included 14 studies; none of them were found by Connected Papers [13]. The research produced 4 results, including the review of interest. In the fourth review, “Nutritional supplementation in the treatment of glaucoma”, 30 papers were selected [14]. Among these, no studies were found by Connected Papers. The research produced 3 results, which included the review of interest. An example of a Connected Papers-generated graph is provided in Figure S1 in Multimedia Appendix 1; it was produced by entering the keywords “glaucoma, pseudoexfoliation, and hearing loss” in the research field and then selecting the first result that appeared on the list.

#### Elicit

The research of relevant records on the first SLR, “Glaucoma pseudoexfoliation and hearing loss,” produced 270 results, which included 15 of 30 studies selected with the PRISMA method for our SLR [11]. Boolean operators were not useful for the research in this platform: none of the 30 previously selected studies were found when we inserted “glaucoma OR PXS OR PXG) AND “hearing loss” in the research box. The second review, “Efficacy of Ahmed and Baerveldt glaucoma drainage device implantation in the pediatric population: A systematic review and meta-analysis,” included 32 studies; 2 papers of the original 32 were identified by Elicit [6,12]. The third review, “Standalone XEN45 Gel Stent implantation in the treatment of open-angle glaucoma: A systematic review and meta-analysis,” included 14 studies, of which 6 were found by Elicit [6,13]. In the fourth review, “Nutritional supplementation in the treatment of glaucoma”, 30 studies were selected by the authors [14]. Among these, 3 papers were found by the Elicit [6]. An example of Elicit’s search results using the keywords “glaucoma pseudoexfoliation and hearing loss” is provided in Figure S2 in Multimedia Appendix 1.

Results of Connected Papers [7] and Elicit [6] research are summarized in Table 1.

**Table 1.** Records found by Connected Papers and Elicit.

	Review 1^a^ (n=30)		Review 2^b^ (n=32)		Review 3^c^ (n=14)		Review 4^d^ (n=30)	
	Connected Papers	Elicit	Connected Papers	Elicit	Connected Papers	Elicit	Connected Papers	Elicit
Selected records found, n (%)	7 (23)	15 (50)	0 (0)	2 (6)	0 (0)	6 (49)	0 (0)	3 (10)

a Review 1: “Glaucoma, Pseudoexfoliation, and Hearing Loss: A Systematic Literature Review” [11].

b Review 2: “Efficacy of Ahmed and Baerveldt glaucoma drainage device implantation in the pediatric population: A systematic review and meta-analysis” [12].

c Review 3: “Standalone XEN45 Gel Stent implantation in the treatment of open-angle glaucoma: A systematic review and meta-analysis” [13].

d Review 4: “Nutritional supplementation in the treatment of glaucoma: A systematic review” [14].

### Data Extraction

#### Elicit

Data extracted from Elicit resulted overall accurate in 655 of 1274 responses (mean 51.4%, SD 31.4%) of cases, with an additional 175 responses (mean 13.7%, SD 18%) correct but lacking necessary and required details (imprecise responses). A total of 285 responses (mean 22.4%, SD 27.5%) were missing, while 159 (mean 12.5%, SD 14.7%) were incorrect.

#### ChatPDF

Data extracted from ChatPDF resulted overall accurate in 768 of 1274 responses (mean 60.3%, SD 30.7%) of cases, with an additional 94 responses (mean 7.4%, SD 13.9%) correct but lacking necessary and required details (imprecise responses). A total of 224 responses (mean 17.6%, SD 20%) of responses were missing, while 187 (mean 14.7%, SD 17.7%) were incorrect.

Figure S3 in Multimedia Appendix 1 illustrates the ChatPDF interface used to extract data from the systematic review studies.

**Figure 1.**
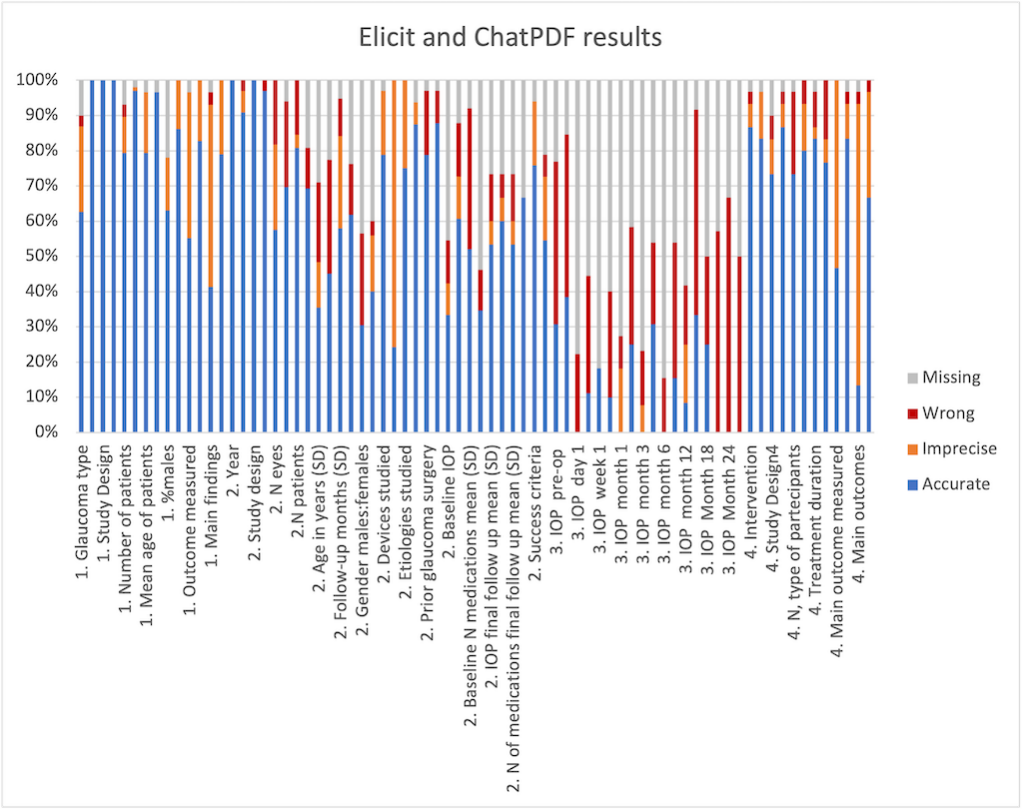
Elicit and ChatPDF data extraction evaluation. Numbers 1‐4 indicate the analyzed review. Review 1: “Glaucoma, Pseudoexfoliation, and Hearing Loss: A Systematic Literature Review”; Review 2: “Efficacy of Ahmed and Baerveldt glaucoma drainage device implantation in the pediatric population: A systematic review and meta-analysis”; Review 3: “Standalone XEN45 Gel Stent implantation in the treatment of open-angle glaucoma: A systematic review and meta-analysis”; Review 4: “Nutritional supplementation in the treatment of glaucoma: A systematic review”. For each variable, the first bar represents Elicit results, and the second bar represents ChatPDF results. IOP: intraocular pressure.

The results of our comparison between the data presented by Elicit, ChatPDF, and the original SLRs are presented in Figure 1.

### AI-Assisted Writing

The two authors (LAM and GM) evaluated Jenni AI’s outputs across multiple dimensions, reaching a consensus on the items under review. The content generated by Jenni AI exhibited satisfactory language fluency, technical proficiency, and suitability. The text produced by Jenni AI was clear, easily understandable, and used appropriate terminology, but it was judged as insufficient for the remaining items. Jenni AI’s number of citations was inadequate, as it only referenced up to three studies throughout its four attempts at writing an SLR. The methods lacked clarity as they did not provide definitions for research terms, specify inclusion and exclusion criteria, or define data extraction and risk of bias assessment. The results section of the study did not provide clear information regarding the number of records identified and excluded, as well as a description of each selected study. In terms of synthesis of results, the study did not present a comprehensive analysis or statistical analysis and did not mention the risk of bias. The discussion section lacked a thorough interpretation of the results. The conclusion paragraph provided a brief overview of the key points covered in the preceding paragraphs, yet it lacked specific details. Each study was typically completed in under 30 minutes, depending on each author’s willingness to revise and rephrase Jenni AI’s writing. To provide a quantitative representation of Jenni AI’s writing performance, the evaluated dimensions are summarized in Table 2. Each item was scored on a scale of 1=poor to 5=excellent. A screenshot of the Jenni AI [9] interface displaying an example of the generated text and features is provided in Figure S4 in Multimedia Appendix 1.

**Table 2.** Quantitative evaluation of Jenni AI’s SLR^a^ writing performance.

Dimension	Score (1-5)	Evaluation
Language fluency	4	Satisfactory: clear and coherent text with appropriate language use, minor grammatical issues.
Technical proficiency	4	Satisfactory: proper use of terminology with acceptable structure, but lacking depth in analysis.
Relevance and suitability	3	Moderate: content is generally on-topic but occasionally lacks focus or depth in specific sections.
Citations	1	Insufficient: limited citations (up to 3) across all drafts, failing to meet SLR standards.
Methods definition	2	Weak: lacks clarity in defining research terms, inclusion or exclusion criteria, and methodology.
Results	2	Weak: insufficient detail on identified or excluded records, and lacks comprehensive data reporting.
Discussion	2	Weak: minimal interpretation of results, lacking critical analysis or synthesis.
Conclusions	3	Vague: summarizes key points but lacks specific insights.

a SLR: systematic literature review.

## Discussion

### Summary of Study Objectives and Findings

This study evaluated the capabilities and limitations of AI tools in the SLR writing process, comparing their performance to the established PRISMA framework [3]. Using four glaucoma-related reviews as case studies, we focused on three key steps in the SLR process: records identification, data extraction, and study composition. We found that while AI tools like Elicit, Connected Papers, ChatPDF, and Jenni AI can streamline certain steps in the SLR process, they exhibit notable gaps in accuracy, completeness, and methodological rigor when compared to the PRISMA framework [3].

### Records search

Starting from the records’ research and selection, we noticed that neither Connected Papers nor Elicit was able to provide the totality of the results found using the PRISMA method. In addition, Elicit’s filters for the papers’ selection were limited and sometimes not precise (for instance, some reviews would be included in the results even though they were excluded in the filters). The given results still needed to be reviewed by an expert, since many of them did not relate to the topic of interest. Many attempts were necessary to understand how to perform the research, in terms of the input mode of keywords (ie, Boolean Operators were not useful to find more studies of interest using both Elicit and Connected Papers); these platforms do not provide instructions on how to correctly perform the research to make it more effective. In regard to Connected Papers, we found that the visual representation of the studies can provide a more direct approach to research and helps with the selection of the most relevant papers. The most cited and most recent papers can be easily recognized immediately thanks to the graphic differentiation of studies by color and size. Regarding SLR, it may be necessary to do more than one research and build various graphs to find the totality of the records present in the literature. This study specifically focused on the Identification stage of the PRISMA [3] framework, representing a limited application of the full process. While PRISMA [3] encompasses a multistep, iterative approach including Screening, Eligibility, and Inclusion, we deliberately constrained our evaluation to the Identification phase. This allowed us to assess the standalone capabilities of AI tools in replicating this initial step and determine if they can streamline the literature search by reducing the need for extensive manual refinement. However, we acknowledge that further research is necessary to evaluate AI tools’ performance across all PRISMA [3] stages, including screening and eligibility assessments.

### Data Extraction

Summaries given by Elicit were still far from how an SLR should be written since they were based on only a few studies (less than 10). Nevertheless, we found the Elicit function of sorting information in the form of tables useful, since it facilitates the understanding and the extrapolation of data and saves time in the process of elaborating the results. The possibility to customize columns was very helpful to obtain the wanted information. Nevertheless, the accuracy of answers was still far from accurate, which indicates the impossibility of relying entirely on this tool to create tables for SLRs. ChatPDF can be used to easily search specific information within a paper, making it easier for the author to quickly access the data of interest without the need to read the entire paper. However, it was not able to reproduce the tables contained in the SLRs object of study, even after providing the software-specific instructions. Additionally, when addressing a folder of studies, the software was unable to answer many questions but could do so when dealing with individual PDFs. This results in a more time-consuming process for generating tables, as each paper needs to be examined individually. As a result, it is not as efficient as Elicit, which can produce the necessary tables much faster. Nevertheless, ChatPDF often provides more accurate results, enhancing its reliability in many cases. As for Elicit, the precision of responses provided by this tool remained inadequate for the purpose of an SLR. While we used structured and targeted queries to maximize the tool’s performance (eg, asking for specific details such as year of publication and baseline intraocular pressure), we opted not to use advanced prompting strategies like iterative rephrasing or chain-of-thought techniques. This decision was made to ensure that the tools were evaluated under practical, real-world conditions and not under artificially optimized scenarios. The observed variability in the accuracy of AI tools can be largely attributed to two key factors: the complexity of the datasets and tool-specific limitations. Studies with straightforward reporting of results in the main text generally yielded higher accuracy, as the tools could easily interpret their structure. In contrast, more complex studies, where critical information was embedded in tables, graphs, or figures, were less effectively processed. These findings highlight the inherent challenges AI tools face when extracting nuanced data from unstructured or nontextual elements. To address these issues, it is critical to align tool selection with the nature of the data being analyzed. Future research should also explore methods to enhance the adaptability of AI tools for more complex datasets, potentially through training or algorithm optimization. Despite these limitations, the variability observed in our results reflects real-world use cases, reinforcing the importance of setting realistic expectations for AI-assisted systematic reviews.

### AI-Assisted Writing

In regard to Jenni AI writing assistant, we found that its suggestions aid in expediting and streamlining the writing process, while also helping prevent “writer’s block.” The paper can be customized based on the author’s preferences by selecting the options among the AI commands, making the draft unique. Nevertheless, the author should, in any case, have mastery of the topic of interest to be able to choose the best option among the ones proposed by the AI, as well as guide the writing in the right direction. Moreover, results showed that there is a need for more systematic and detailed methods in Jenni AI’s literature review process. The paper written by Jenni AI lacked accurate and exhaustive results, which were supported by too few citations; moreover, the discussion of results was vague. The paper written by Jenni was overall inadequate and did not meet the requirements of an SLR. As a result, we believe that Jenni AI can be helpful in assisting writers in text paraphrasing or summarization, but its limitations in accuracy and completeness indicate that it should not be solely relied upon for generating high-quality content.

We acknowledge that this study was specifically focused on glaucoma-related SLRs, as our expertise in this domain enabled a critical evaluation of AI tools’ outputs. However, this narrow scope may limit the generalizability of our findings, and further research across diverse fields is necessary to validate the applicability of these tools more broadly.

Nonetheless, there are other aspects to be taken into consideration: conducting a comprehensive review of academic literature allows us to generate our own research concepts, thus enhancing our ability for critical analysis and originality [17]. Engaging in bibliographical research is crucial for developing critical analysis skills, establishing academic identity, identifying gaps and areas for further exploration, and contributing to the advancement of our research field; these frameworks contribute to ongoing discussions about thoughts and methodologies with those who came before us. Leaving this role entirely to AI may result in us missing the core objective of performing research, which is to actively engage in and enrich existing knowledge through critical evaluation [17]. Furthermore, we think that authors should specify in their papers if AI was used in the SLR writing process, in order to provide transparency and clarity regarding the methodology of their work. Indeed, many journals ask authors to disclose in their studies the use of AI and AI-assisted technologies [18].

### Conclusions

Our research showed that the PRISMA method continues to exhibit clear superiority in terms of reproducibility and accuracy at three key steps of conducting an SLR, starting from the search of relevant records, through the collection and organization of data, up to the writing of the final paper. Although its contribution can save time and help with repetitive tasks, it is important to note that AI lacks critical thinking, and expert analysis continues to be necessary to identify potential biases in the algorithms. For this reason, researchers must approach AI-generated content with a thoughtful and discerning mindset, recognizing the benefits while also acknowledging the need for careful scrutiny. While this study focused on glaucoma research due to our domain-specific expertise, its findings highlight the potential and limitations of AI tools in SLRs. Further validation across diverse domains and datasets is needed to enhance their applicability. As AI continues to advance, it holds promise for providing support and improvement to the SLR process. However, at present, human oversight and refinement are still crucial for the comprehensive and accurate completion of SLRs. Therefore, it is imperative that while leveraging AI, researchers should continue to apply their expertise and critical thinking skills to ensure the quality, accuracy, objectivity, and integrity of their work.

## Supplementary material

10.2196/68592 Multimedia Appendix 1 Supplementary figures.

## Abbreviations

AI: artificial intelligence
LLM: large language model
PRISMA: Preferred Reporting Items for Systematic reviews and Meta-Analyses
SLR: systematic literature review

## References

[1] Levels of evidence (march 2009). Oxford Centre for Evidence-Based Medicine Levels of Evidence.

[2] Karger E, Jagals M, Ahlemann F (2021). Blockchain for smart mobility—literature review and future research agenda. Sustainability.

[3] Page MJ, Moher D, Bossuyt PM (2021). PRISMA 2020 explanation and elaboration: updated guidance and exemplars for reporting systematic reviews. BMJ.

[4] Yu P, Xu H, Hu X, Deng C (2023). Leveraging generative AI and large language models: a comprehensive roadmap for healthcare integration. Healthcare (Basel).

[5] Thirunavukarasu AJ, Ting DSJ, Elangovan K, Gutierrez L, Tan TF, Ting DSW (2023). Large language models in medicine. Nat Med.

[6] (2024). Elicit: The AI research assistant. Elicit.

[7] Find and explore academic papers. Connected Papers.

[8] (2024). Chat with any PDF. ChatPDF.

[9] Meet your intelligent research assistant. Jenni AI.

[10] Ammar W, Groeneveld D, Bhagavatula C Construction of the literature graph in semantic scholar.

[11] Meliante LA, Piccotti G, Tanga L, Giammaria S, Manni G, Coco G (2024). Glaucoma, pseudoexfoliation and hearing loss: a systematic literature review. J Clin Med.

[12] Stallworth JY, O’Brien KS, Han Y, Oatts JT (2023). Efficacy of Ahmed and Baerveldt glaucoma drainage device implantation in the pediatric population: a systematic review and meta-analysis. Surv Ophthalmol.

[13] Lim SY, Betzler BK, Yip LWL, Dorairaj S, Ang BCH (2022). Standalone XEN45 Gel Stent implantation in the treatment of open-angle glaucoma: a systematic review and meta-analysis. Surv Ophthalmol.

[14] Loskutova E, O’Brien C, Loskutov I, Loughman J (2019). Nutritional supplementation in the treatment of glaucoma: a systematic review. Surv Ophthalmol.

[15] PubMed.

[16] Embase.

[17] Guersenzvaig A, Sánchez-Monedero J (2025). AI research assistants, intrinsic values, and the science we want. AI Soc.

[18] The use of generative AI and AI-assisted technologies in writing for Elsevier: policy for book and commissioned content authors. Elsevier.

