# Supplementary material for: Evaluation of AI Tools Versus the PRISMA Method for Literature Search, Data Extraction, and Study Composition in Glaucoma Systematic Reviews: Content Analysis
Source: JMIR AI. 2025 Sep 5;4:e68592. doi: 10.2196/68592 (PMC12413140; doi:10.2196/68592)

Figure S1. Example of Connected Papers-generated graph.


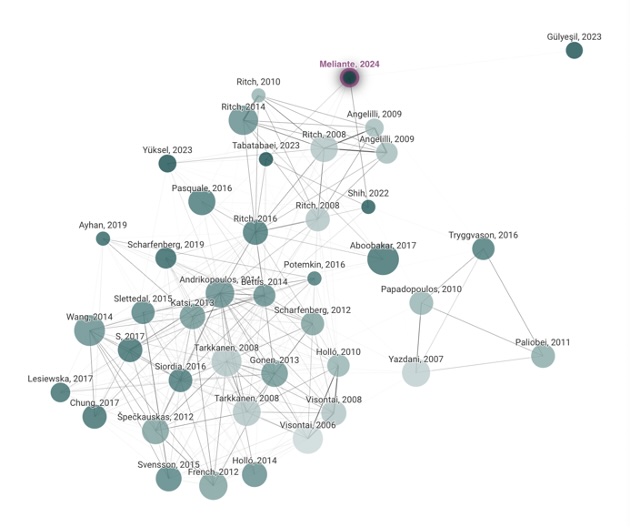


Figure S2. Example of Elicit’s search results using the keywords “glaucoma pseudoexfoliation and hearing loss.”


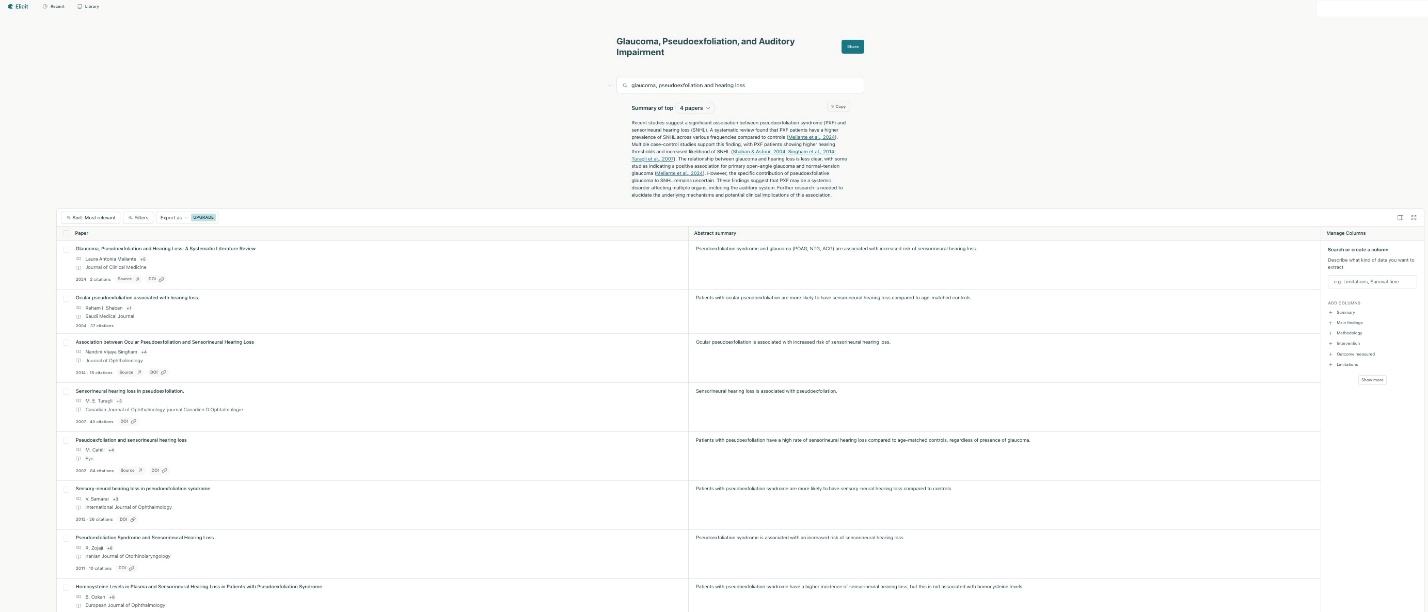


Figure S3. ChatPDF interface used to extract data from the systematic review studies.


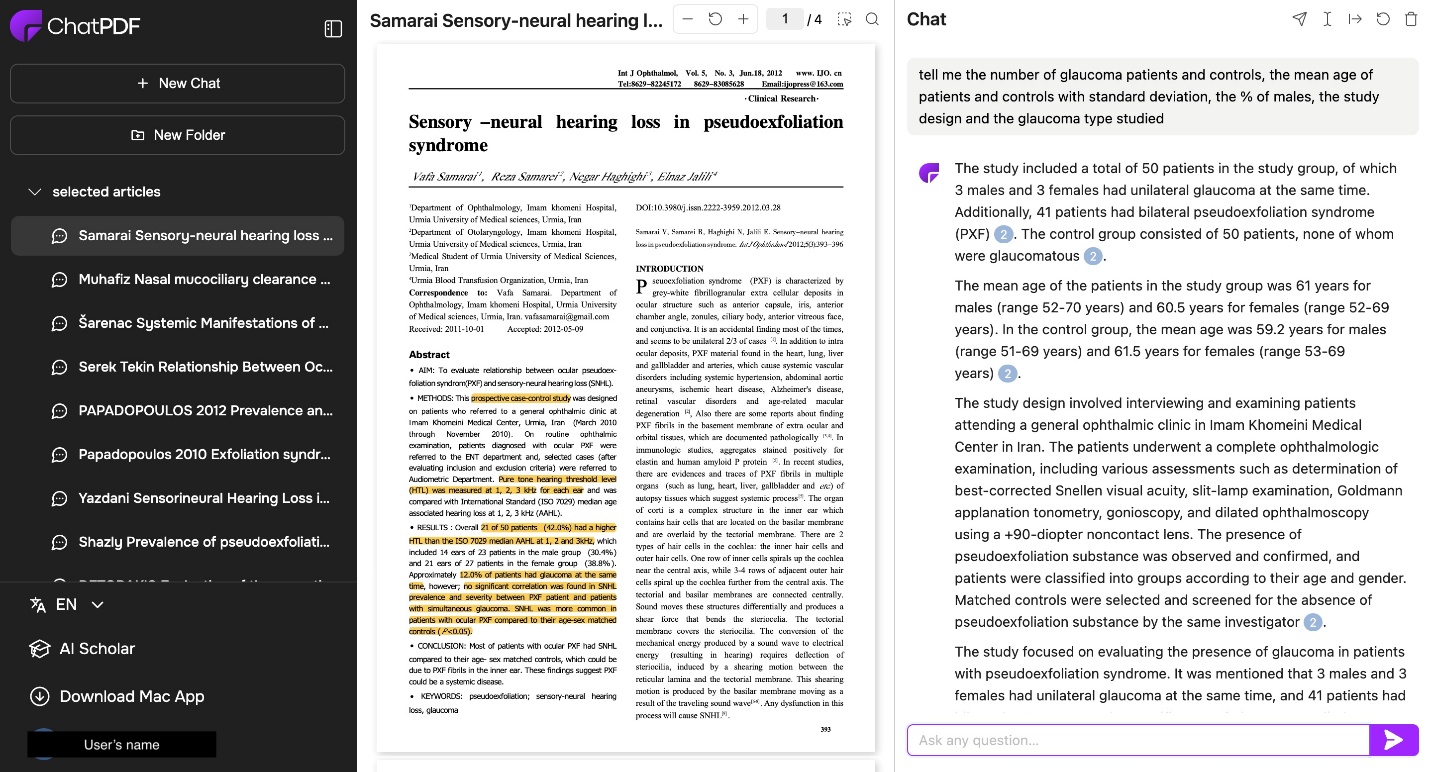


Figure S4. Screenshot of the JenniAI interface displaying an example of the generated text and features.
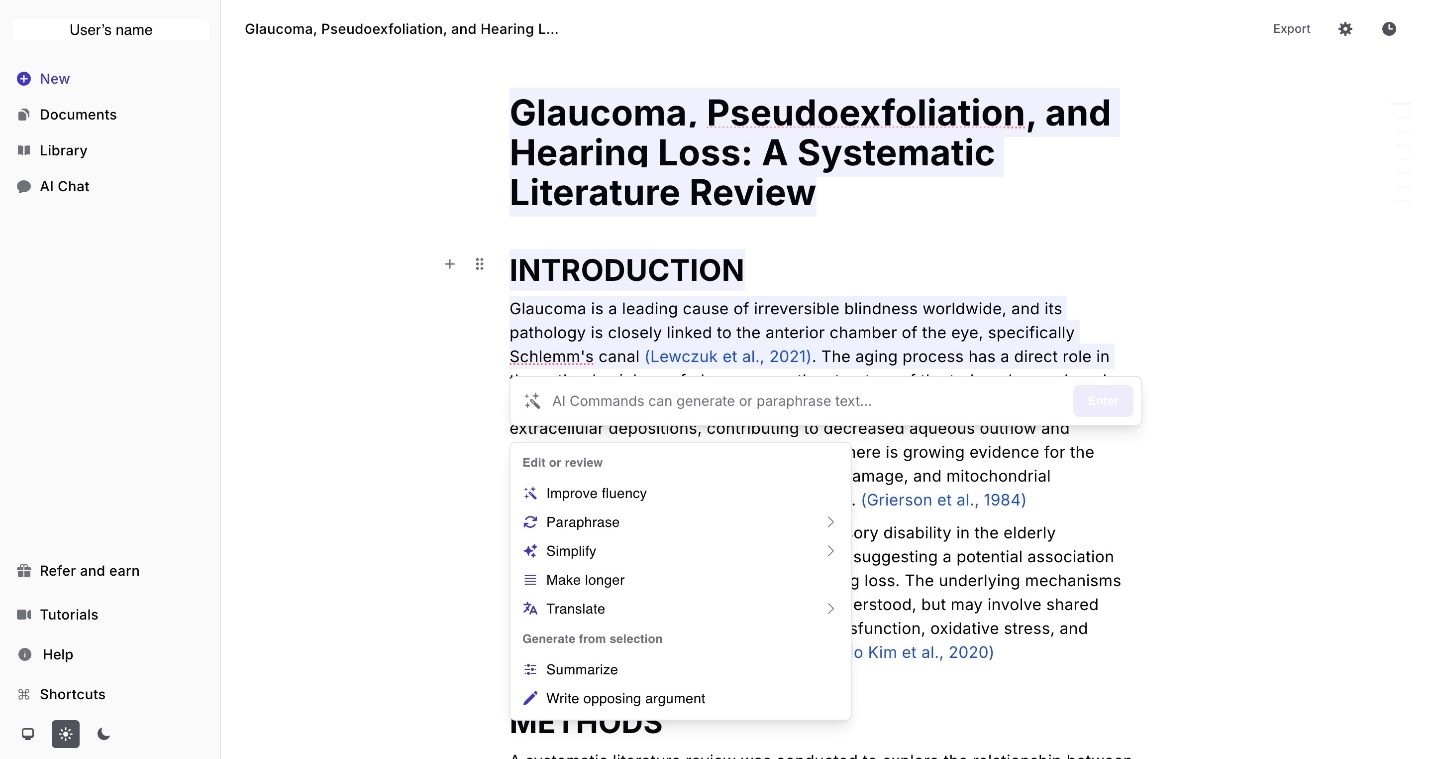

Supplement: Multimedia Appendix 1 [file ai-v4-e68592-s001.docx]
